# The *Caenorhabditis elegans* voltage-gated calcium channel subunits UNC-2 and UNC-36 and the calcium-dependent kinase UNC-43/CaMKII regulate neuromuscular junction morphology

**DOI:** 10.1186/1749-8104-8-10

**Published:** 2013-05-10

**Authors:** Raymond C Caylor, Yishi Jin, Brian D Ackley

**Affiliations:** 1 Department of Molecular Biosciences, University of Kansas, 5004 Haworth Hall, 1200 Sunnyside Ave, Lawrence, KS, 66045, USA; 2 Neurobiology Section, Division of Biological Sciences, University of California at San Diego, La Jolla, CA, 92093, USA; 3 Howard Hughes Medical Institute, Chevy Chase, MD, 20815, USA

**Keywords:** Calcium channels, Synaptogenesis, Extracellular matrix, Nidogen, LAR-RPTP, α-liprin, Cell adhesion, Calmodulin kinase II

## Abstract

**Background:**

The conserved *Caenorhabditis elegans* proteins NID-1/nidogen and PTP-3A/LAR-RPTP function to efficiently localize the presynaptic scaffold protein SYD-2/α-liprin at active zones. Loss of function in these molecules results in defects in the size, morphology and spacing of neuromuscular junctions.

**Results:**

Here we show that the Ca_v_2-like voltage-gated calcium channel (VGCC) proteins, UNC-2 and UNC-36, and the calmodulin kinase II (CaMKII), UNC-43, function to regulate the size and morphology of presynaptic domains in *C. elegans*. Loss of function in *unc-2, unc-36* or *unc-43* resulted in slightly larger GABAergic neuromuscular junctions (NMJs), but could suppress the synaptic morphology defects found in *nid-1/*nidogen or *ptp-3/*LAR mutants. A gain-of-function mutation in *unc-43* caused defects similar to those found in *nid-1* mutants. Mutations in *egl-19,* Ca_v_1-like, or *cca-1,* Ca_v_3-like, α1 subunits, or the second α2/δ subunit, *tag-180,* did not suppress *nid-1*, suggesting a specific interaction between *unc-2* and the synaptic extracellular matrix (ECM) component nidogen*.* Using a synaptic vesicle marker in time-lapse microscopy studies, we observed GABAergic motor neurons adding NMJ-like structures during late larval development. The synaptic bouton addition appeared to form in at least two ways: (1) *de novo* formation, where a cluster of vesicles appeared to coalesce, or (2) when a single punctum became enlarged and then divided to form two discrete fluorescent puncta. In comparison to wild type animals, we found *unc-2* mutants exhibited reduced NMJ dynamics, with fewer observed divisions during a similar stage of development.

**Conclusions:**

We identified UNC-2/UNC-36 VGCCs and UNC-43/CaMKII as regulators of *C. elegans* synaptogenesis. UNC-2 has a modest role in synapse formation, but a broader role in regulating dynamic changes in the size and morphology of synapses that occur during organismal development. During the late 4th larval stage (L4), wild type animals exhibit synaptic morphologies that are similar to those found in animals lacking NID-1/PTP-3 adhesion, as well as those with constitutive activation of UNC-43. Genetic evidence indicates that the VGCCs and the NID-1/PTP-3 adhesion complex provide opposing functions in synaptic development, suggesting that modulation of synaptic adhesion may underlie synapse development in *C. elegans.*

## Background

Changes in the cell membrane potential can open voltage-gated calcium channels (VGCC) to permit calcium entry. VGCCs are formed by α1, α2/δ, and β subunits and can include a γ subunit [1]. The α1 subunit forms the channel pore, while the auxiliary β and α2/δ subunits affect channel trafficking and physiology [2,3]. The α2/δ subunits are synthesized as a single polypeptide that undergoes proteolytic cleavage, but remains covalently associated [4-6]. VGCCs have been classified by their pharmacological and electrophysiological properties [1]. Ca_v_2-type channels are localized to the presynaptic active zone, where they function in vesicle exocytosis. Ca_v_1 channels localize more broadly and have been associated with events including gene regulation, local translation and dendritic growth [7].

In addition to synaptic vesicle exocytosis, there have been reports describing a role for VGCCs in regulating presynaptic development. Loss of calcium signaling through the α1 subunit *cacophony* resulted in reduced neuromuscular junction (NMJ) growth during Drosophila larval development [8]. At the vertebrate NMJ, a role for synaptic calcium channels has been discovered in mediating synaptic stability. The Ca_v_2.1 and Ca_v_2.2 calcium channels were found to bind directly to the extracellular matrix proteins laminin-10 and −11 and this interaction is critical for synaptic maintenance [9]. In the central nervous system, the α2/δ1 protein is a receptor for the extracellular matrix (ECM) protein thrombospondin, and together they act to promote synaptogenesis [10]. These results demonstrate how an interaction between VGCCs and the synaptic ECM can regulate synaptic development and morphology.

Previously, we have shown the synaptic ECM components nidogen (*nid-1)* and collagen XVIII (*cle-1)* exhibit distinct roles in the formation of *Caenorhabditis elegans* NMJs [11]. Mutations in *nid-1* cause a disruption in the size, shape and function of these synapses by disrupting the accumulation of the LAR receptor tyrosine phosphatase, PTP-3A, and the intracellular-adaptor protein α-liprin/SYD-2 at active zones [12].

During a screen for *nid-1* genetic modifiers we recovered an allele of *unc-2,* which encodes the single *C. elegans* Ca_v_2-like α1 subunit [13]. Loss-of-function (LOF) mutations in *unc-2* result in presynaptic contacts being slightly enlarged, but also in suppression of the defects caused by the *nid-1* mutation. Using time-lapse analysis we show that GABAergic NMJs exhibit dynamic shape changes during the late 4th larval stage (L4), and that new NMJs can be formed by the elongation and division of established presynaptic domains. These dynamic changes were dependent on functional UNC-2. We also find that *unc-43,* the *C. elegans* Calmodulin kinase II (CaMKII) homolog, regulates GABAergic synapse formation. LOF in *unc-43* suppresses *nid-1,* while a gain-of-function (GOF) mutation in *unc-43* causes *nid-1-*like defects in NMJ morphology. Our results find a novel interaction between the ECM and VGCCs, where, during synaptic development, they appear to function antagonistically.

## Results and discussion

### *unc-2* regulates the size, shape and morphology of presynaptic domains

**Figure 1:**
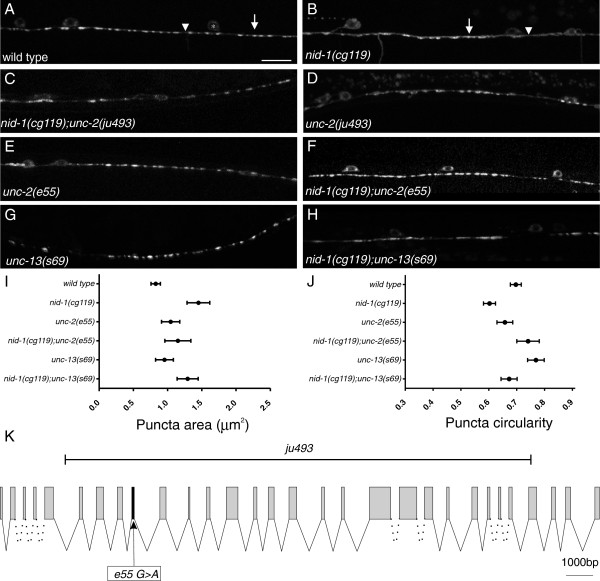
***unc-2 *mutations suppress SNB-1::GFP defects in *nid-1*.** (**A**) In wild type animals synaptic vesicles accumulated in evenly spaced puncta (arrow) with smooth morphology (arrowhead). Cell bodies (asterisks) are also visible. Scale bar equals 10 μm. (**B**) Animals lacking nidogen accumulated synaptic vesicles in elongated (arrow) puncta. Diffuse GFP was often present in regions between puncta (arrowhead). (**C**) The *ju493* mutation suppressed the large aggregations of SNB-1::GFP seen in *nid-1* mutants. (**D**, **E**) Mutations in *unc-2* (D - *ju493*, E - *e55*) resulted in slightly larger puncta, but the morphology and distribution were more similar to wild type than *nid-1*, even when *nid-1* was absent (**F**). (**G**) *unc-13(s69)* animals had a slight decrease in the number of puncta, but the morphology was not grossly affected. (**H**) In the *nid-1(cg119);unc-13(s69)* double mutants puncta were elongated similar to *nid-1* single mutants. (**I, J**) We quantified puncta area (K) and circularity (L) for each analyzed genotype and plotted them as mean (circle) with the 5 to 95% confidence interval (whiskers). (**K**) A cartoon of the genomic region of *unc-2* with the position of alleles used indicated. Exon/introns are to scale, except for dashed lines, which indicate larger introns. The *e55* allele is a G > A nucleotide change in exon 9 that results in Q571 to stop [Wormbase: CE42155]. The *ju493* deletion removes a large part of the coding segment, corresponding to the exons encoding AA144-1888. All animals in these micrographs are young adults (1 to 2 days post 4th larval stage (L4)).

We have found that the ECM protein nidogen, *nid-1,* affects the morphology and function of *C. elegans* NMJs [11,12]. We visualized GABAergic NMJs using a SNB-1::GFP (synaptobrevin) chimeric marker, *juIs1*[14], as an indicator of presynaptic size and placement. In young adult (yAd) wild type (wt) animals GFP-labeled synaptic vesicles cluster in discrete, regularly sized puncta that have a smooth morphology (Figure 1A), with an average area of 0.81 ± 0.01 μm^2^ (Mean ± S.E.M.). Strong LOF *nid-1(cg119)* animals have a synapse defective (Syd) phenotype such that fluorescent puncta often exhibit a rough or disorganized appearance and have an apparent area of 1.45 ± 0.08 μm^2^*P* <0.05) (Figure 1B).

To better understand how nidogen affects NMJs, we conducted a screen for genetic modifiers of the *nid-1* phenotype (see Methods). We recovered *ju493*, which appeared to largely suppress the synaptic vesicle accumulation defects present in *nid-1(cg119)* animals (Figure 1C). By themselves, the *ju493* animals were uncoordinated (Unc), and also displayed synaptic morphology defects (Figure 1D). We mapped the allele to the left arm of linkage group X, and found *ju493* failed to complement *unc-2(e55)*, a strong LOF allele [13]. By PCR amplification and sequence analysis, we found that *ju493* animals had a large deletion within the *unc-*2 coding region, suggesting that it is likely a strong LOF allele (Figure 1K). *unc-2* is one of three *C. elegans* genes encoding VGCC α1 subunits. We tested LOF alleles in the other two VGCC, *egl-19* and *cca-1*, and did not observe any suppression of *nid-1* defects [See Additional file 1: Figure S1], indicating the observed effect is specific to *unc-2.*

We further characterized the effect of *unc-2* LOF using the *e55* allele, which causes a premature stop codon in exon 9 (Figure 1K). Presynaptic domains of *unc-2(e55)* mutants had smooth and oval-shaped SNB-1::GFP puncta, but the area of the puncta was significantly increased over wt by approximately 20% (1.04 ± 0.07 μm^2^, *P* <0.05) (Figure 1E) in yAd animals. We also found that the total number of presynaptic clusters was slightly reduced in *unc-2(e55)* mutants (wt 25.6 ± 0.2 puncta/100 μm versus *unc-2* 22.8 ± 0.7 puncta/100 μm (*P* <0.05)). Like *ju493*, the *e55* mutation suppressed vesicle-accumulation defects found in *nid-1(cg119)* (Figure 1F). Puncta formed in *nid-1;unc-2* double mutants had an average area of 1.15 ± 0.10 μm^2^ (*P* <0.05 versus *nid-1* or wt*; P* >0.05 versus *unc-2*), and appeared morphologically similar to wt.

To more quantitatively assess the NMJ morphology, we calculated the circularity of the SNB-1::GFP fluorescent puncta (Figure 1J) (see Methods). Wild type SNB-1::GFP puncta are ovoid, and have an average circularity of 0.70 ± 0.01. By comparison, in *unc-2(e55)* the puncta varied slightly, but significantly, from wt (0.66 ± 0.03, *P* <0.05), while in *nid-1* mutants, the puncta were more elongated and less circular (0.60 ± 0.01, *P* <0.05). The *nid-1;unc-2* double mutants were like wt (0.74 ± 0.02, *P* <0.05 versus *nid-1* or *unc-2; P* >0.05 versus wt), indicating that the elongated morphology in *nid-1* was ameliorated by removing *unc-2* function.

UNC-2 is the single Ca_v_2-like VGCC α1 subunit present in *C. elegans,* and is localized to the presynaptic active zone [15]. At synapses UNC-2 regulates calcium ion entry that facilitates synaptic vesicle exocytosis [16]. It is the reduced neural transmission from motor neurons to muscles that likely results in the reduced locomotor activity and the Unc phenotype. To address that the suppression of the *nid-1* presynaptic defects was not the result of reduced exocytosis and/or locomotor activity, we tested a LOF mutation in *unc-13,* which plays essential roles in synaptic transmission and also regulates the sub-synaptic accumulation of synaptic vesicles [16,17]. *unc-13(s69)* null mutants are also Unc, but have a more severe loss of locomotor activity than *unc-2.* In *unc-13(s69)* yAd animals, puncta were slightly, but not significantly, enlarged (0.95 ± 0.07 μm^2^, *P* >0.05 versus wt) (Figure 1G), and there was a significant reduction in the number of puncta formed (18.7 ± 1.5/100 μm (*P* <0.05 versus wt). In *nid-1(cg119);unc-13(s69)* double mutants, presynaptic domains were *nid-1* like, elongated and disorganized (1.29 ± 0.10 μm^2^, *P* >0.05 versus *nid-1* and *P* <0.05 versus *unc-13*) (Figure 1H). This result suggests that the suppression of *nid-1* by *unc-2* is unlikely to be the result of impaired exocytosis or reduced locomotion.

We identified *unc-2* as a regulator of presynaptic development because it largely suppressed the morphological defects present in *nid-1* LOF animals. Ca_v_2-like channels are known to affect synaptic development [8,9], including by linking synapses to the ECM [18,19]. In *Drosophila* mutations in *cacophony*, a Ca_v_2 α1 voltage-gated calcium channel subunit, result in fewer NMJs being formed, although loss of syntaxin or synaptobrevin showed normal synapses, arguing the loss of the calcium channel had an effect on synapses distinct from its role in exocytosis [8]. We find that although *unc-13* mutants have fewer presynaptic domains, this effect was insufficient to suppress the morphological defects caused by the loss of *nid-1.* It has also been shown that GABA, the neurotransmitter used by the neurons examined here, is not required for NMJ formation [20,21]. From these results we conclude that UNC-2 regulates presynaptic development in *C. elegans* independently from neurotransmission, and the phenotype caused by the loss of NID-1 at synapses requires functional UNC-2.

### Presynaptic domains exhibit developmentally dynamic morphologies

**Figure 2:**
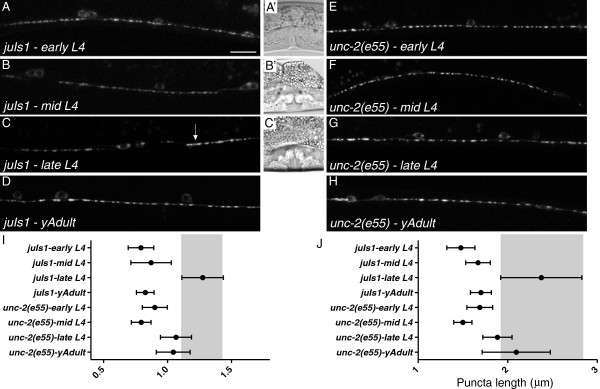
**Wild type** (**wt) animals have enlarged puncta during late 4th larval stage (L4) development.** During the early (**A**) and mid **(B**) L4 stage SNB-1::GFP puncta in wild type animals were evenly sized and spaced. **(C**) But during late L4 puncta were often enlarged and/or elongated (arrow) along the axon. **(A**’**-C**’) Representative examples of vulval progression used to mark transition from early (**A**’), mid (**B**’) and late (**C**’) phases of L4 development. (**D**) In young adults puncta were again evenly sized and spaced. In *unc-2* mutants the early (**E**) and mid (**F**) L4 stage animals had evenly sized and spaced puncta, that appeared enlarged in late L4 (**G**), but not elongated. (**H**) Puncta remained enlarged in young *e55* adults, compared to those in equivalently aged wild type animals (**D**). (**I**) Plots of the puncta length in the different stages are presented as means (circles) with the 5 to 95% confidence intervals (whiskers). During late L4 of wild type (area also shaded for comparison), puncta are longer, and more variable in length, than during other periods imaged. By comparison, during late L4, although longer than wild type adults, *unc-2* mutants had only a modest increase in length. (**J**) A plot of the length of each puncta divided by the width for each genotype.

*C. elegans* development consists of four larval stages, ultimately leading to an approximately 5 to 10-fold increase in organism length. Since no new GABAergic motor neurons are added after the first larval stage, axons of these neurons, to accommodate the increase in body size, must grow and add synapses accordingly. Since all previous observations were made in yAd animals, we examined synaptic puncta of wt animals in the early, mid and late L4 stages, using the maturation of the vulva as a guide for the developmental stage [22]. In the early L4 stage, SNB-1::GFP puncta in GABAergic neurons appeared of normal size (0.79 ± 0.05 μm^2^) (Figure 2A). The average area of the puncta noticeably increased starting during the mid-L4 stage (0.87 ± 0.08 μm^2^) and through the late-L4 stage (1.27 ± 0.08 μm^2^), and the puncta shapes were frequently found to be elongated, often with a disorganized perimeter (Figure 2B, C). Elongated, disorganized puncta were infrequently observed in yAd stage animals (0.81 ± 0.01 μm^2^) (Figure 2D), indicating that in wt*,* presynaptic domains have developmentally dynamic morphologies.

*unc-2* mutants displayed fewer presynaptic domains than wt animals (Figure 1). We wondered if this was an effect of the failure to establish synaptic domains during development or the deterioration of existing connections. We found that the puncta in early (0.90 ± 0.05 μm^2^) and mid L4 (0.79 ± 0.04 μm^2^) were evenly sized and regularly shaped (Figure 2E, F). However, in late L4 *unc-2(e55)* animals, enlarged puncta were observed, although to a lesser degree than wt (1.07 ± 0.06 μm^2^)*,* but did not appear disorganized. Similarly sized puncta were observed in young *unc-2* adults (1.04 ± 0.07 μm^2^) (Figure 2H).

**Figure 3:**
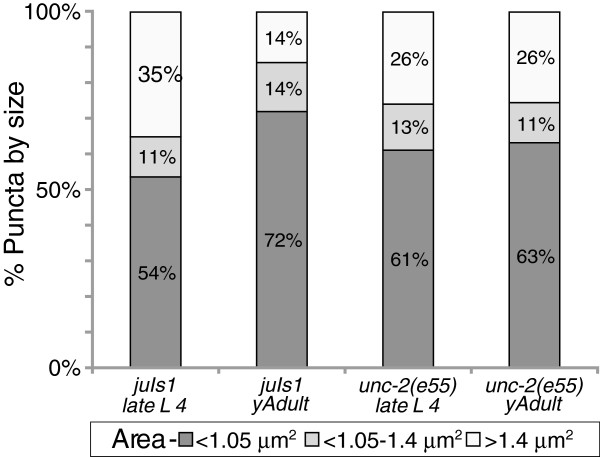
**Presynaptic domains reshape during the 4th larval stage (L4) to adult transition.** The proportion of puncta with an enlarged shape was analyzed. Puncta were binned into three categories based on the area [(<1.05, wild type like), (1.05-1.40, *unc-2* like) and (>1.40, *nid-1* like)]. During late L4 in wild type 35% of the puncta were enlarged and appeared *nid-1* like, compared to only 14% in young adults. In contrast, the percent of puncta in *unc-2* animals in any of the categories was not changed during the L4 developmental stage. N >150 puncta/genotype/stage.

To assess the frequency of enlarged puncta relative to the total number of presynaptic domains observed, we binned individual puncta measured into categories, based on measured area (Figure 3). During the late L4 stage, approximately 35% of the puncta in wt animals appeared enlarged, with an area greater than 1.4 μm^2^ (approximately *nid-1*-like), compared to yAds, where less than 15% of puncta fit into that category. The increased number of enlarged puncta came at the expense of normal-sized puncta, suggesting a natural size change occurs during this period. By comparison, there was no change in the proportion of the population (26%) of puncta that measured larger than 1.4 μm^2^ in *unc-2(e55)* when we compared late L4s to yAds (Figure 3).

**Figure 4:**
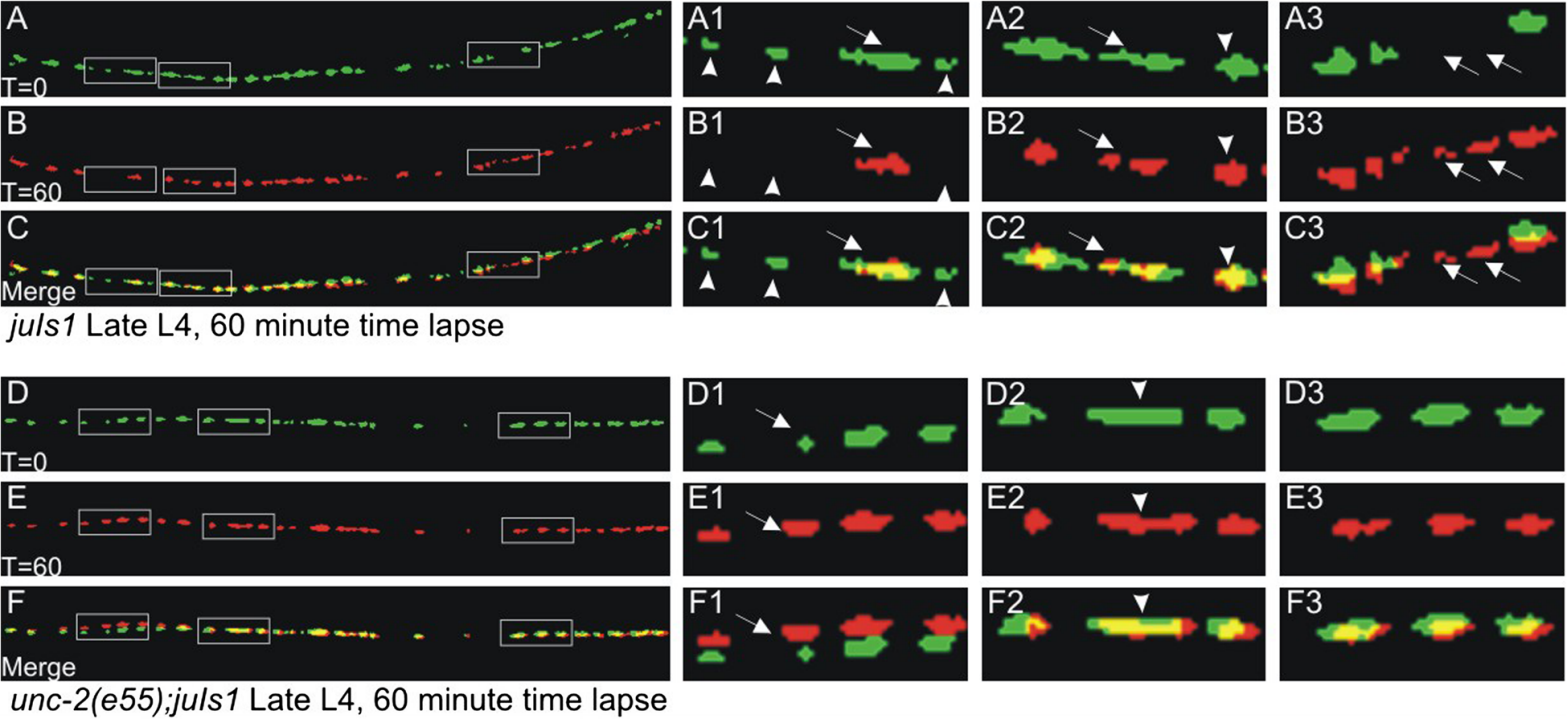
***unc-2 *mutants exhibit fewer dynamic neuromuscular junctions (NMJs) during the 4th larval (L4) stage.** SNB-1::GFP puncta formed in the ventral cord of wild type or *unc-2(e55)* animals were imaged for one hour. The panels are a mask of the threshold image and false colored to demonstrate changes in the shape of the puncta. In wild type after one hour the puncta largely align (yellow), with some differences, highlighted in the boxed regions. (**A-C1**) Three small puncta (arrowheads) that are present at time 0 have disappeared 60 minutes later (B1). The elongated punctum (arrow) has condensed. (**A-C2**) An elongated punctum present at time 0 (arrow) appears to have separated into two distinct puncta. An adjacent punctum (arrowhead) is largely unchanged. (**A-C3**) An empty space at time 0 has two puncta (arrows) that have formed. (**D-F**) In *unc-2* mutants puncta generally enlarged, but we observed fewer formation/elimination and division events. (**D-F1**) One punctum (arrow) was approximately twice as large after 60 minutes, while the adjacent puncta were largely unchanged. Note: the animal moved slightly during the protocol, putting the puncta in D1-F1 slightly out of alignment. (**D-F2**) We found occasional puncta that appeared to be elongated (arrowhead). In *unc-2* these puncta remained elongated after an hour whereas in wild type they often resolved into two puncta, or shrunk in size. (A-C2). (**D-F3**) Many SNB-1::GFP puncta were approximately the same size, undergoing little change during the time lapse, indicating that SNB-1::GFP was not affected by photobleaching over the imaging protocol.

To investigate more precisely the dynamic changes in synapse morphology during the late-L4 stage, we performed time-lapse confocal microscopy on live, non-anesthetized animals (see methods). In wt animals, we saw the following dynamic behaviors: SNB-1::GFP puncta forming *de novo,* puncta that disappeared, puncta that changed shape by increasing and decreasing in size, and at a low frequency, an existing punctum divided such that two puncta were generated (Figure 4A-C). Overall, per segment of the nerve cord examined (approximately 150 to 200 μm), a net increase of 1 puncta/hour in wt animals was observed.

By contrast in *unc-2(e55)* animals we found little evidence of dynamic shape changes. Puncta still formed *de novo,* and puncta did enlarge, but rarely did those enlarged puncta shrink or divide (Figure 4D-F) as was seen in wt. Overall, per segment of the nerve cord examined *unc-2* mutants displayed a net loss of approximately 3 NMJ puncta/hour under our time-lapse protocol. It is not clear whether these were budding events that initiated and failed, or were delayed. The decreased number of puncta seen by GFP imaging may be due to a failure in *unc-2* animals to add and/or maintain synapses during development. Alternatively, our observations are also consistent with a significant delay in synaptic development, although not a total failure of synaptic addition. Due to the presence of *de novo* appearance of SNB-1::GFP puncta in *unc-2(e55)* mutants during the time-lapse imaging, we infer the observed synaptic dynamics likely relies on UNC-2 function after, not during, initial NMJ formation. Further, preliminary analyses indicate that synapses formed in the L3 stage of *unc-2* animals can also be enlarged, indicate the function of UNC-2 is not confined to the L4 developmental stage.

### *unc-36* genetically interacts with *unc-2* to regulate neuromuscular junction morphology

**Figure 5:**
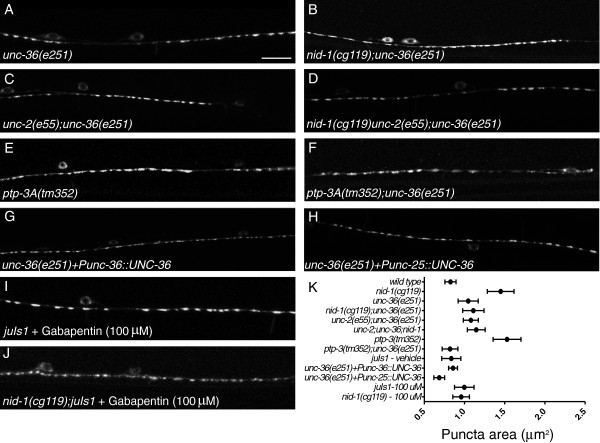
***unc-36 *regulates presynaptic morphology*.*** (**A, B**) *unc-36* mutants also had slightly enlarged puncta, and also suppressed the defects in *nid-1.* (**C**) *unc-2;unc-36* mutants appeared similar to the single mutants, but were not further enlarged. (**D**) Suppression of *nid-1* was not enhanced by simultaneous loss of both *unc-2* and *unc-36.* (**E**) A deletion in the *ptp-3A* specific coding region, *tm352,* causes a *nid-1* like defect in synapses, which is also suppressed by loss of *unc-36* (**F**). (**G**) Broadly replacing UNC-36 function via transgene efficiently rescued the enlarged puncta found in *unc-36(e251)* mutants. (**H**) Specific expression of UNC-36 solely in the D-type motor neurons was also sufficient to reduce the puncta enlargement found in *unc-36(e251)***.** (**I, J**) Treatment of wild type animals (I) or *nid-1(cg119)* (J) with the α2/δ-subunit antagonist, gabapentin, resulted in enlarged puncta similar to those found in *e251.* (**K**) The puncta area by genotype are plotted as the mean (circle) with 5 to 95% confidence interval (whiskers). All animals in these micrographs are young adults (1 to 2 days post 4th larval stage (L4)).

Mutations in *unc-36,* one of two VGCC α2/δ subunit genes in the *C. elegans* genome, often phenocopy *unc-2*[15,23,24], so we tested if *unc-36* was required for normal synaptic morphology. Similar to *unc-2, unc-36(e251)* caused an increase in SNB-1::GFP area in yAd animals (1.10 ± 0.07 μm^2^) (*P* <0.05) (Figure 5A). A slight, but not significant, decrease in puncta number (23.1 ± 1.5) (*P* >0.05) was also observed. *unc-36(e251);nid-1(cg119)* double mutants appeared like *unc-36* synapses alone, with an apparent area of 1.11 ± 0.07 μm^2^ (*P* <0.05 compared to *nid-1(cg119)* and *P* >0.05 versus *unc-36(e251)*) (Figure 5B). Double mutants of *unc-2* and *unc-36* had puncta that resembled each of the single mutants (1.08 ± 0.05 μm^2^, *P* >0.05 versus *unc-2* or *unc-36* alone) (Figure 5C). *unc-2(e55);unc-36(e251);nid-1(cg119)* triple mutants displayed no significant differences from either of the double mutant combinations (1.15 ± 0.06 μm^2^, *P* >0.05 versus *unc-2;nid-1* or *unc-36;nid-1*) (Figure 5D). *unc-36* was also able to suppress *ptp-3A* defects (1.50 ± 0.09 μm^2^ (*P* <0.05) versus *unc-36(e251);ptp-3A(tm352)* = 0.80 ± 0.10 μm^2^ (*P* <0.05) (Figure 5E,F). From these data, we concluded *unc-2* and *unc-36* likely function in a linear genetic pathway to interact with *ptp-3A* and *nid-1* in synapse development.

We also tested the second *C. elegans* α2/δ subunit-encoding gene, *T24F1.6*/*tag-180*[25]. A LOF allele, *ok779,* exhibited a minor effect on SNB-1::GFP area (0.73 ± 0.03 μm^2^ (*P* <0.05)) [see Additional file 1: Figure S1], and number (28.8 puncta/100 μm (*P* >0.05)) in yAd animals. *tag-180(ok779);nid-1(cg119)* double mutants were more like *nid-1(cg119)* than the *ok779* (1.65 ± 0.12 μm^2^, *P* <0.05 versus *nid-1* alone), indicating the suppression of *nid-1* defects is specific to *unc-36*. Double mutants of *ok779* with *unc-2* (1.02 μm^2^, *P* >0.05 versus *unc-2* alone) or *unc-36* (1.14 μm^2^, *P* >0.05 versus *unc-36* alone) similarly showed no significant change from the *unc-2* or *unc-36* single mutants.

Next we addressed whether the role of *unc-36* is cell autonomous. We were able to rescue the Unc (not shown) and Syd (Figure 5G) defects in yAd *unc-36(e251)* animals by reintroducing a wild type copy of UNC-36 under the control of the endogenous promoter (N = 4 lines) (puncta area - 0.84 ± 0.03, *P* <0.05 versus *unc-36(e251)*). The *unc-36* promoter is broadly active in the animal, including the muscles and nervous system [15,25,26]. To determine whether *unc-36* could be functioning cell autonomously*,* we specifically expressed UNC-36 in the GABAergic motor neurons in *unc-36(e251);juIs1* animals. As expected, the locomotor defects were not rescued, but the size of the presynaptic puncta in these animals was significantly reduced relative to *unc-36(e251)* yAd animals lacking the transgene (0.69 ± 0.05, *P* <0.05 versus *unc-36(e251)*) (Figure 5H) (N = 1 line), indicating that *unc-36* is capable of cell autonomously affecting the GABAergic NMJs.

α2/δ subunits have been implicated in synapse formation and development [10,27,28]. In the vertebrate central nervous system (CNS), thrombospondin (TSP) molecules act as ligands for α2/δ1. Overexpression of α2/δ1 results in increased synaptogenesis, while interfering with the TSP-α2/δ1 interaction inhibited synaptogenesis. The C-terminal region of TSP, that contains EGF repeats, binds directly to the von Willebrand factor-A domain (VWF-A) present in the α2 portion of the α2/δ1 protein [10]. Since NID-1 contains EGF repeats and localizes near NMJs [11,29], and UNC-36 has a VWF-A domain, it is possible that NID-1 might physically interact with UNC-36. However, at GABAergic NMJs, the loss of *nid-1* causes morphological defects that are suppressed by removing *unc-36.* Also, LOF in *unc-36* has a very modest reduction in synapses formed. Thus, it seems unlikely that NID-1 acts as a ligand for UNC-36 to promote synapse addition.

There is evidence from *Drosophila* that α2/δ subunits can have effects on synaptic development independent from the α1 subunits [28]. However, our data suggest *unc-36* and *unc-2* are acting in the same genetic pathway. Work from the Bargmann lab has demonstrated that UNC-36, along with the calcium channel chaperone CALF-1, is required for the localization of UNC-2 to the synaptic plasma membrane [15]. Thus, a simple explanation for the synaptic patterning defects would be a failure to direct UNC-2 to the synaptic plasma membrane in *unc-36(e251)* mutants. In this model, we would assume that UNC-2 is the key player required to drive changes in synaptic development, although we cannot rule out UNC-36 may have additional functions at NMJs.

### *unc-36* function is required during the 4th larval stage period for synaptic development

Next, to address the temporal requirement of VGCC, we used the α2/δ-antagonist gabapentin [30] to acutely inhibit VGCC function specifically during the L4 stage of development. In cultured vertebrate neurons gabapentin inhibits the trafficking of α2/δ subunits from the endoplasmic reticulum to the plasma membrane [31]. Wild type early L4 animals were exposed to gabapentin (100 μM) for 24 hours. When imaged as yAds, these animals had a significant increase in SNB-1::GFP area (1.00 ± 0.06 μm^2^, *P* <0.05 versus vehicle alone - 0.83 ± 0.06) (Figure 5I). This suggests that gabapentin is phenocopying *unc-36* LOF, although since presynaptic domains in double mutants of *unc-36* with *tag-180* resembled those found in *unc-36* alone, we cannot rule out that gabapentin was broadly affecting α2/δ function.

Gabapentin treatment of *nid-1(cg119)* mutant L4 animals, resulted in a significant decrease in the SNB-1::GFP area (0.96 ± 0.05 μm^2^, *P* <0.05 versus *nid-1* alone), indicating that acute gabapentin treatment of *nid-1(cg119)* animals during the L4 stage was able to, at least partially, suppress presynaptic defects (Figure 5J). We conclude from our analysis that the synaptic patterning that occurs in late L4 animals requires intact α2/δ function during that period, rather than being fixed in an earlier developmental event. *nid-1* mutants have defective presynaptic morphologies prior to L4, and these appear to be ameliorated by LOF in *unc-2.* Thus, we do not rule out a function for VGCCs prior to L4, and this result would suggest that synaptic morphologies are broadly dynamic during L4 development.

### CaMKII regulates synaptic morphology

**Figure 6:**
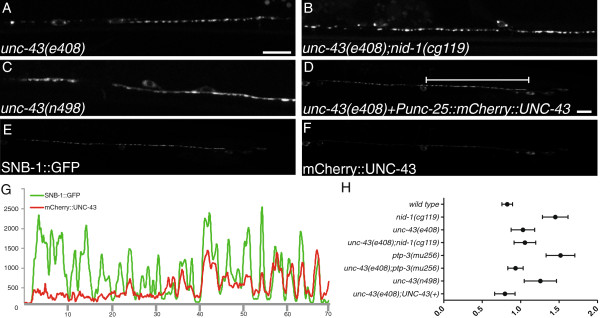
***unc-43 *can cell autonomously regulate presynaptic morphology.** (**A**) The *unc-43(e408)* loss-of-function (LOF) mutation caused an increase in puncta size*.* (**B**) The *e408* mutation suppressed the morphological defects found in the *nid-1* background. (**C**) The *n498* gain-of-function (GOF) mutation in *unc-43* caused enlarged puncta that were disorganized. (**D-F**) Expression of an mCherry-tagged UNC-43 chimera specifically in the GABAergic neurons largely rescued the SNB-1::GFP morphology defects present in *e408* animals. (**G**) A line scan of the VD12 axon region (line in panel D) demonstrating the co-incidence of mCherry::UNC-43 (red) with SNB-1::GFP (green). GFP/RFP coincidence was measured in a single confocal slice. (**H**) A plot of the puncta area measured by genotype as mean (circle) with the 5 to 95% confidence interval (whiskers). All animals in these micrographs are young adults (1 to 2 days post 4th larval stage (L4)).

To identify molecules that might utilize changes in local calcium concentrations to shape presynaptic domains, we undertook a candidate-molecule approach. The first gene we examined was *unc-43*/CaMKII. CaMKII has been extensively linked to both pre- and postsynaptic development in multiple organisms (for review see [32,33]). UNC-43 has been shown to work downstream of *unc-*2, and UNC-43 localization is dependent on UNC-2. CaMKII proteins have been shown to affect synapse development in multiple systems, including *C. elegans* where UNC-43 functions in the synaptic development of the glutamatergic neurons [34-36]. Because the complete loss of *unc-43* is embryonic lethal, we used a hypomorphic LOF allele, *e408. e408* yAd animals have slightly enlarged SNB-1::GFP (Figure 6A) (1.03 ± 0.08 μm^2^, *P* <0.05 versus wt). Thus, reduced UNC-43 function has a similar manifestation at synapses as the loss of UNC-2 and UNC-36.

Like LOF in *unc-2* or *unc-36,* the *unc-43(e408)* LOF suppressed defects present in *nid-1* (Figure 6B) (1.06 ± 0.07 μm^2^, *P* <0.05 versus *nid-1*) and *ptp-3A* yAds (*ptp-3A(ok244)* (1.03 ± 0.12 μm^2^, *P* <0.05 versus *ptp-3A(ok244)* (1.42 ± 0.09 μm^2^)). In contrast, *unc-43(n498)* GOF [37]*,* bearing a constitutively activating mutation, E108K, in the active site core [35], SNB-1::GFP puncta were elongated and disorganized in yAds (1.26 ± 0.11 μm^2^, *P* <0.05 versus wt) (Figure 6C), and similar to *nid-1* (*P* >0.05 versus *nid-1*).

We next examined whether *unc-43* was functioning cell autonomously by expressing an mCherry-tagged *unc-43* cDNA, under the control of the *unc-25* promoter, in the *unc-43(e408);juIs1* animals. Replacing UNC-43D (Wormbase: CE28054) specifically in the GABAergic neurons rescued the defects in *e408,* (0.83 ± 0.05 μm^2^, (N = 6 lines) *P* >0.05 versus wt and *P* <0.05 versus *e408*). The RFP::UNC-43 chimera was localized throughout the cytoplasm, including being present at synapses, as evidenced by co-localization with the SNB-1::GFP (Figure 6D-G). The localization of UNC-43 suggests it could be directly functioning at NMJs to locally affect morphology.

## Conclusions

### VGCCs contribute to synaptic development in *Caenorhabditis elegans*

Here we show that mutations in the single Ca_v_2, *unc-2,* result in changes to the number, size and shape of presynaptic domains that form during development. The overall reduction in the number of synapses formed in *unc-2* mutants is modest, suggesting that the primary role of VGCCs is not to promote *de novo* synapse addition. Rather, UNC-2 function is most evident in the maintenance of presynaptic domain morphology, as *unc-2* mutants display areas that are enlarged relative to wild type.

The function of *unc-2* in regulating presynaptic morphology is also observed when the synaptically associated adhesion molecules NID-1 or PTP-3A are absent. Removing UNC-2 from *nid-1* or *ptp-3* loss-of-function backgrounds ameliorates the mislocalization of presynaptic proteins and synaptic overgrowth in those mutants. This indicates that the morphological changes associated with the loss of NID-1-mediated adhesion require functional UNC-2, but independent of synaptic vesicle exocytosis.

### Regulation of adhesion and growth at synapses

Synapses are dynamic structures that can be added or removed, or change shape to accommodate functional changes in neural networks and/or organismal growth. In Drosophila, transient changes in adhesion have been shown directly to permit switching between synaptic stability and synaptic growth [38-43]. Molecularly, both Fas2 and *discs large* (DLG) appear to be important for synaptic stability. Down regulation of Fas2-mediated adhesion by activation of CaMKII has been shown to specifically induce synaptic growth [40,42,43].

DLAR, the Drosophila PTP-3 homolog, has also been shown to complex with different ligands to switch between synaptic stability and synaptic growth [39]. In vertebrates, cell adhesion molecules like laminins and nidogens are required for NMJ maintenance. In cultured vertebrate neurons, regulation of liprin-α, via CaMKII, has been shown to regulate LAR to affect dendritic spine stability at excitatory synapses [40]. Overall, these findings suggest that changes in synaptic morphology that occur normally in development require modulation of the synaptic adhesion that maintains synaptic structure.

Using time-lapse analysis we find that, in *C. elegans,* during a specific developmental window, presynaptic domains in wild type animals can be morphologically similar to those found in *nid-1/ptp-3A* mutant adults. Further, we observed that a *nid-1*-like elongated punctum could divide to form multiple new puncta. These dynamics, both elongation and division, were dependent on *unc-2.* Based on our results and data from other systems, a simple model is that nidogen-LAR adhesion maintains NMJ structure, and that developmental changes in synaptic morphology require transient inhibition of nidogen-LAR adhesion, and that this occurs via a pathway that includes UNC-2, UNC-36 and UNC-43.

### Cooperative versus antagonistic interactions between the extracellular matrix and voltage-gated calcium channels in synaptic maintenance

Previous work has shown that the ECM and the VGCCs work cooperatively to stimulate synapse development [9,10,19]. For example, the synaptically associated laminin β2 subunit can directly bind to an extracellular loop in the VGCC α1 polypeptide. In cultured neurons, this interaction induces the clustering of synaptic vesicles at the binding site. However, neither the genetic ablation of either Ca_v_2.1 or laminin β2, nor disrupting the binding of these proteins *in vivo* results in a total failure in synapse formation [9]. Rather, a defect in synaptic growth and/or maintenance was observed in those animals. Similarly, NMJs in mice lacking nidogen-2 do form normally, but fail to develop, beginning to fragment around three weeks after birth, suggesting a role in synaptic maintenance for nidogen-2 as well [44], although no interaction has been described between nidogen and VGCCs in vertebrates.

We have found that nidogen is required for synaptic maintenance, which suggests a conservation of function for this ECM molecule. However, we also see that the defects observed in *nid-1* mutants require functional UNC-2 VGCCs. Thus, in contrast to the apparent cooperative interaction observed in vertebrates, our study suggests perhaps an antagonistic interaction between VGCCs and ECMs in *C. elegans*. It is possible that our findings reflect a difference in the growth of vertebrate NMJs that form at axon terminals and *C. elegans* NMJs that form *en passant*.

It is worth noting that we also find that presynaptic domains in *unc-2* mutants are slightly enlarged in comparison to wild type animals. This may seem contradictory in that the loss of *unc-2* also limits synapses from elongating during specific periods of development or when *nid-1* is absent. A simple way of thinking about this is that in the absence of UNC-2, NMJs are too stable, unable to respond to signals that instruct them to enlarge or shrink. This would be a novel finding for this class of VGCCs: that they function as key regulators of dynamic changes in synaptic morphology. Going forward our goal will be to identify how UNC-2 can regulate these seemingly distinct functions at NMJs.

## Methods

### *Caenorhabditis elegans* strains

All *C. elegans* strains were maintained at 20 to 22.5°C as described [45]. The following alleles were used in this report: N2 (var. Bristol), *nid-1(cg119), unc-2(ju493), unc-2(e55), unc-36(e251), tag-180(ok779), egl-19(n582), cca-1(ad1650), ptp-3A(tm352)*, *ptp-3A(tm352); ptp-3A(ok244), ptp-3(mu256), unc-43(n498), unc-43(e408), nid-1(cg119)rpm-1(ju44), unc-13(s69), tra-2(q276)/mnC1*. The following integrated strain was used: *juIs1* [*Punc-25*::SNB-1::GFP]. Transgenic animals were generated by germ line transformation as described [46]. To conduct the *ju493/e55* non-complementation, *tra-2(q276);juIs1/+;ju493/+* XX males were crossed to *unc-2(e55)* hermaphrodites. Cross progeny were identified by presence of GFP (*juIs1*), non-complementation was determined by presence of UncGfp animals.

### Genetic modifier screen

*nid-1(cg119)rpm-1(ju44);juIs1* animals were mutagenized using 50mM ethane methyl sulfonate (EMS). F2 animals were scored for a hypercontracted uncoordinated phenotype that is observed in *syd-2;rpm-1* double mutants. Individual HypUnc animals were allowed to self-fertilize to insure transmission of the phenotype. The animals were outcrossed to either *nid-1(cg119)* or *rpm-1(ju44)* single mutant backgrounds to determine the effect of new mutations.

### Image analysis

Synapse morphology of D type neurons was visualized by *juIs1* [*Punc-25* SNB-1::GFP]. All images were collected on either a Zeiss Pascal confocal microscope or an Olympus FV1000 confocal microscope equipped with Fluoview software (Olympus America Inc., Center Valley, PA USA). Images were acquired using multi-track parameters when necessary (*unc-36* and *unc-43* rescue), with either a 63X or 60X Plan-apochromat objective, respectively. Animals were anesthetized using 0.5% phenoxy-propanol (TCI America, Portland, OR U.S.A.) in M9 and mounted on 2% agarose pads. Measurements of SNB-1::GFP were as described with minor modifications [11]. All images were collected using the exact same microscope settings. Briefly, confocal images were projected into a single plane using the maximum projection and exported as a tiff file with a scale bar. Using ImageJ the files were converted to a binary image using the threshold command, so that the binary image resembled the RGB image. A region of interest was drawn around the relevant region of the nerve cords. The following measurement options were selected: Area, Center of Mass, Circularity, Perimeter, Fit Ellipse, and Limit to Threshold. Scaling was set by measuring the scale bar. The “Analyze Particle” command was used with a minimum of four pixels and no maximum size. The following options were selected: Outline Particles, Ignore Particles Touching Edge, Include Interior Holes and Reset Counter. The resulting measurements were exported to Microsoft Excel and GraphPad Prism for statistical analysis. Comparisons of single mutants to the wild type were tested by Students two-tailed *t*-test, while double and triple mutant combinations were compared within the group using a Kruskal-Wallis test with a Dunn’s Multiple Comparison post hoc test. Circularity is a measure of how close to a perfect circle an object is, where 0 is a line and 1 is a perfect circle. The test relates the area of the observed object to the area of a circle with the same radius:

$$\left(\text{formula}=4\mathrm{pi}*\left(\text{area}/\text{perimete}r^{2}\right)\right).$$

### UNC-36 rescue

To rescue the *unc-36(e251)* defect we generated a PCR product including the putative promoter and endogenous 3^′^UTR using the following primers (*unc-36promF1: 5*^*′*^*-ccacgtacatagaattcggaatc-3*^′^ and *unc-36 3*^*′*^*UTR R1: 5*^*′*^*- caaggcagttggaaagtcgac-3*^′^). The PCR product was TOPO cloned into pCRXLII (Life Technologies, Grand Island, NY USA) to generate pBA234. pBA234 was injected at 10 ng/μl into *unc-36(e251);juIs1* animals along with pPD118.33 (*Pmyo-2::gfp*) as a co-injection marker, plus pBA186 (*Punc-25::mCherry)* to mark the GABAergic motor neurons containing the transgene.

### Cell-specific rescue

A genomic fragment covering the *unc-36* coding region was amplified using the following primers (*unc-36promF1: 5*^*′*^*-ccacgtacatagaattcggaatc-3*^*′*^ and *unc-36 3*^*′*^*UTR R1: 5*^′^*- caaggcagttggaaagtcgac-3*^′^). The PCR product was T/A cloned into the pCR8/GW/TOPO vector (Life Technologies), and then was recombined using L/R clonase (Life Technologies) into pBA153, creating pEVL404 (*Punc-25::unc-36).* pEVL404 was injected into *unc-36(e251);juIs1* animals at 10 ng/μl along with pPD118.33 (*Pmyo-2::gfp*) as a co-injection marker. For *unc-43,* we isolated a full-length cDNA for UNC-43D isoform by RT-PCR from wild type RNA isolated by Trizol using the following primers (*unc-43cDNA F1: 5*^*′*^*-atgatgaacgcaagcacca-3*^′^ and *unc-43cDNA R1: 5*^*′*^*- ctagaattcagatactgttgtatttgttg-3*^′^). Using the InFusion (Clontech Laboratories, Inc., Mountain View, CA USA) enzyme this product was recombined into pEVL387 (*Punc-25::mCherry::unc-43 3*^*′*^* UTR*) to generate pEVL400 (*Punc-25::mCherry::unc-43E::unc-43 3′UTR*). The pEVL400 plasmid was injected into *unc-43(e408);juIs1* at 5 ng/μl. Any additional information about sequences or cloning procedures is available upon request.

### Time-lapse analysis

L4 animals were immobilized on 10% agarose pads in the presence of 5% (w/v) polystyrene beads (Bangs Laboratory, Fishers, IN USA). Animals were imaged at 5-minute intervals for 1 hour. Animals that died during the acquisition process (as determined by a rapid and dramatic increase in intestinal autofluorescence) were excluded from the analysis. Images were then exported to ImageJ. Z-stacks were produced for each time point, and then times 0 and 60 minutes were thresholded, converted to masks and overlaid as false colored, green and red respectively. This allowed simple determination of spots that were added or removed, grew or shrank or divided during the analysis period. The total number of puncta added/lost during the hour session was determined by comparing initial time and final time points for the appearance or disappearance of puncta.

### Pharmacology

Gabapentin (Sigma Aldrich, St. Louis, MO USA) was resuspended in DMSO at 100 mM, and then diluted in 1:10 in PBS and added to standard NGM plates seeded with OP50 *E. coli* to achieve the final desired concentration. Using plates with an increasing dose of gabapentin, we found that animals reared throughout development on NGM plates containing 100 μM gabapentin phenotypically resembled *unc-36,* appearing thin, with poor movement (data not shown), thus we used this concentration for our experiments. Plates were permitted to dry overnight and then 20 L4 animals of each genotype were placed on the media. Animals were imaged 24 hours later (as young adults).

## Abbreviations

CaMKII: calmodulin kinase II; CNS: central nervous system; ECM: extracellular matrix; GABA: γ-amino butyric acid; GOF: gain of function; LOF: loss of function; L4: 4th larval stage; NMJ: neuromuscular junction; S.E.M.: standard error of the mean; Syd: synapse defective; Unc: uncoordinated movement; VGCC: voltage-gated calcium channel; wt: wild type.

## Competing interests

The authors declare that they have no competing interests.

## Authors’ contributions

RCC and BDA carried out the molecular, genetic and cell biological studies and collected and analyzed the data. YJ and BDA designed and conducted the genetic screen to isolate *ju493.* BDA drafted the manuscript. All authors have read and approved the final manuscript.

## Supplementary Material

Additional file 1: Figure 1 Specificity of interaction between *unc-2/unc-36* and *nid-1*. **(A)** The *egl-19* LOF allele, *n582*, animals had normal appearing SNB-1::GFP puncta. **(B)** In *egl-19(n582); nid-1(cg119)* the puncta appear enlarged and disorganized. **(C)** A deletion in the Cav3-like channel, *cca-1*, had no obvious effect on SNB-1:GFP morphology. **(D)** Removing *cca-1* from *nid-1* mutants did not suppress the morphological changes, as puncta were observed to be elongated and disorganized. **(E)** Loss of the second ***α***2/***δ*** subunit, *tag-180*, had only a modest effect on SNB-1::GFP puncta, but did not suppress the defects found in *nid-1* mutants **(F)**. **(G)** A plot of the puncta area measured by genotype as mean (circle) with the 5 to 95% confidence interval (whiskers). All animals in these micrographs are young adults (1 to 2 days post larval stage 4 (L4)). Click here for file
